# Functional characterization of the ER stress induced X-box-binding protein-1 (Xbp-1) in the porcine system

**DOI:** 10.1186/1471-2199-12-25

**Published:** 2011-05-24

**Authors:** Jin-Yu Zhang, Kyu-Sun Lee, Ji-Su Kim, Bong-Seok Song, Dong-Il Jin, Deog-Bon Koo, Kweon Yu

**Affiliations:** 1 Aging Research Centre, Korea Research Institute of Bioscience and Biotechnology (KRIBB), Daejeon 305-806, Korea; 2 Dept. Animal Science, Chungnam National University, Daejeon, Korea; 3 National Primate Research Center, KRIBB, Ochang, Chungbuk Korea; 4 Dept. of Biotechnology, Daegu University, Gyeongbuk, Korea

## Abstract

**Background:**

The unfolded protein response (UPR) is an evolutionary conserved adaptive reaction for increasing cell survival under endoplasmic reticulum (ER) stress conditions. X-box-binding protein-1 (Xbp1) is a key transcription factor of UPR that activates genes involved in protein folding, secretion, and degradation to restore ER function. The UPR induced by ER stress was extensively studied in diseases linked to protein misfolding and aggregations. However, in the porcine system, genes in the UPR pathway were not investigated. In this study, we isolated and characterized the porcine *Xbp1 *(*pXbp1*) gene in ER stress using porcine embryonic fibroblast (PEF) cells and porcine organs. ER stress was induced by the treatment of tunicamycin and cell viability was investigated by the MTT assay. For cloning and analyzing the expression pattern of *pXbp1*, RT-PCR analysis and Western blot were used. Knock-down of *pXbp1 *was performed by the siRNA-mediated gene silencing.

**Results:**

We found that the *pXbp1 *mRNA was the subject of the IRE1α-mediated unconventional splicing by ER stress. Knock-down of *pXbp1 *enhanced ER stress-mediated cell death in PEF cells. In adult organs, *pXbp1 *mRNA and protein were expressed and the spliced forms were detected.

**Conclusions:**

It was first found that the UPR mechanisms and the function of pXbp1 in the porcine system. These results indicate that pXbp1 plays an important role during the ER stress response like other animal systems and open a new opportunity for examining the UPR pathway in the porcine model system.

## Background

Endoplasmic reticulum (ER) is an essential cellular compartment for protein synthesis and maturation [1]. The perturbation of ER functions such as disruption of Ca^++ ^homeostasis, inhibition of protein glycosylation or disulfide bond formation, hypoxia, and bacteria infections results in the accumulation of unfolded or mis-folded proteins in ER lumen. To reduce the excessive mis-folded protein loading, cells trigger unfolded protein response (UPR) including the transient attenuation of protein translation, the degradation of mis-folded proteins, and the induction of molecular chaperones and protein folding enzymes [1]. In severe ER stress, UPR results in cell death through the activation of apoptotic pathways [2].

Three distinct UPR signaling pathways exist in mammalian cells that include ER transmembrane inositol-requiring enzyme 1 (IRE1), PKR-like ER kinase (PERK), and activating transcription factor 6 (ATF6) pwthways [3]. The evolutionary conserved IRE1 in the UPR pathway plays as a Ser/Thr protein kinase and endoribonuclease [4,5]. Upon the activation of IRE1α by ER stress, the endonuclease domain of IRE1 splices the *XBP1 *mRNA and removes 26 base pairs from the full-length *XBP1 *mRNA by the unconventional splicing. This event results in the conversion of the premature unspliced XBP1 protein (XBP1u, 267 amino acids) to the spliced XBP1 protein (XBP1s, 371 amino acids) by the frame shift. XBP1s induces a subset of UPR target genes related to protein quality control, ER translocation, glycosylation, and ER/Golgi biogenesis [1,6].

UPR has been intensively studied in various model systems including mice and human cell lines. However, the UPR pathway and its components have not yet been elucidated in the porcine model system. Pigs are an important resource in biomedical research because they are more similar to humans than the rodent model [7,8]. Therefore, pigs have wide implications for studying human diseases such as diabetes obesity, hypertension, and cardiovascular diseases [9-11]. In addition, transgenic pigs are used as the bioreactor for the production of various growth hormones used in human medicine [12]. Organs from cloned pigs produced by the somatic cell nuclear transfer (SCNT) can be used in xenotransplantation [13-15].

In this study, we report the function of pXbp1, the porcine ortholog of the human Xbp1, in ER stress. The unconventional splicing of *pXbp1 *mRNA is evolutionary conserved. In the RNA interference-mediated *pXbp1 *knock-down, we found that the *pXbp1 *participated in ER stress-mediated cell death through the regulation of the target gene expression. We also detected the spliced *pXbp1S *mRNA and protein in adult organs of pigs.

## Results

### Tunicamycin induces UPR in PEF cells

**Figure 1:**
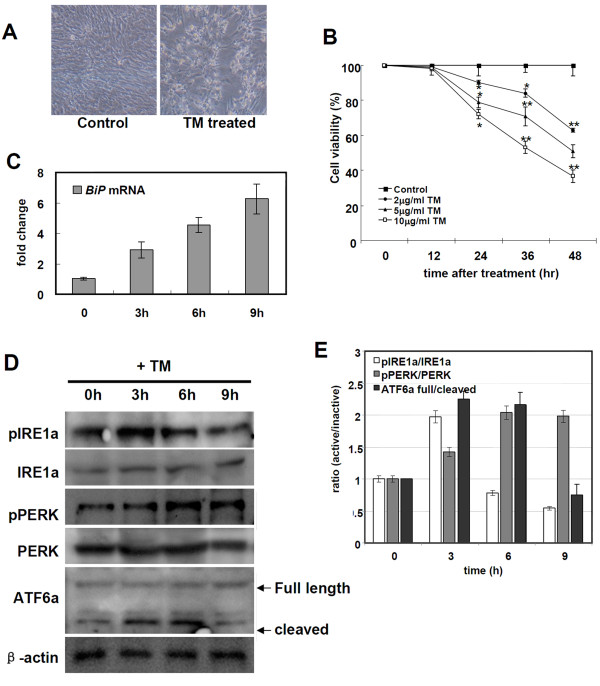
**Tunicamycin treatment induced cell death and unfolded protein response in PEF cells**. (A) Tunicamycin treatment for 24 h in PEF cells induced cell death compared to the control PEF cells and (B) the cell death was dose-dependent. Data are presented as means ± SEM. *P < 0.05, **P < 0.001. (C) Tunicamycin treatment increased expression of an ER stress marker BiP in the RT-PCR analysis. (D) Three major ER stress sensors IRE1, PERK, and ATF6 were activated after TM treatment. (E) Quantification of the Western blot analysis in (D). Data were presented as means ± SD from three independent experiments.

Tunicamycin (TM), which inhibits N-linked glycosylation in newly synthesized polypeptide, induces ER stress [16]. We tested whether the treatment of TM triggers ER stress-induced cell death in porcine embryonic fibroblast (PEF) cells. When cells were treated with TM for 24 h, we detected the morphological changes and the reduction of cell numbers (Figure 1A). The effect of TM on ER stress-mediated cell death was also detected by the morphological changes of apoptotic nuclei and the activity of caspase-3 (Additional file 1, Fig. S1). In addition, the cell viability was significantly decreased from 12 h in TM treated cells. The cell death was time and dose-dependent (Figure 1B).

To access whether ER stress response is activated in PEF cells by the TM treatment, we measured the expression levels of a ER stress marker BiP [17] and the activation of three major ER stress sensors IRE1α, PERK, and ATF6 with the Western blot analysis. BiP protein expression was significantly up-regulated from 3 h (Figure 1C). IRE1α and ATF6 were activated from 3 h and PERK was activated from 6 h after TM treatment (Figure 1D). These results indicate that ER stress induces unfolded protein response in PEF cells.

### Cloning and sequence analysis of porcine Xbp1

**Figure 2:**
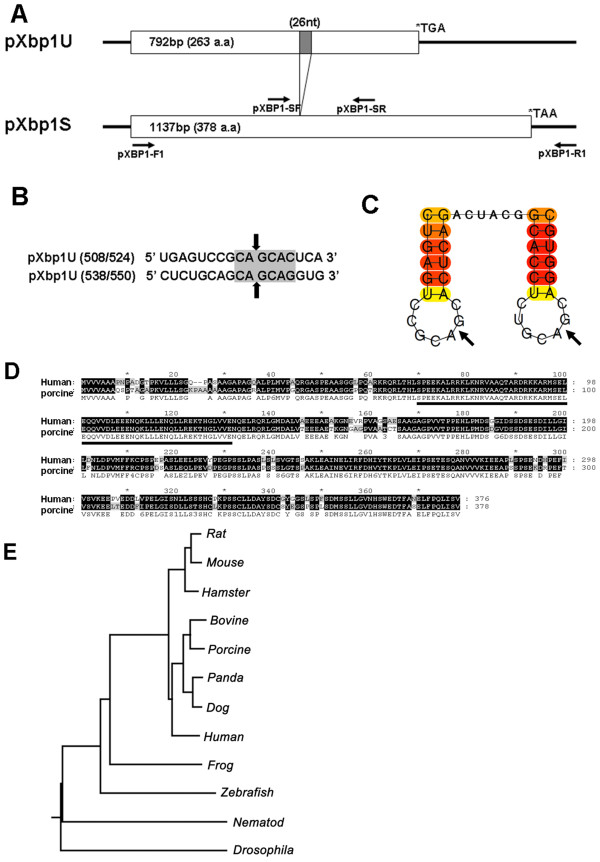
**Sequence analysis of pXbp1**. (A) After IRE1-mediated splicing, 26 nucleotides were deleted from unspliced *pXbp1U *mRNA and produced the 378 amino acids spliced pXbp1 protein. Arrow shows the primers used in this study. pXbp1-F1 and pXbp1-R1 were used to amplify the full length of *pXbp1 *mRNA. pXbp1-SF and pXbp1-SR were used to detect the spliced *pXbp1 *mRNA. (B) The consensus sequence of the cleavage targets by IRE1α is in gray and the cleavage sites are indicated by arrows. (C) Schematic representation of the cleavage sites with secondary structure were predicted by CentroidFOLD. (D) Amino acid sequence comparisons between the human XBP1 and porcine XBP1 protein using the ClustalW method. Identical amino acid residues are shaded in black and the basic leucine zipper (bZIP) domain is underlined. (E) Phylogenetic trees of XBP1 were generated with the neighbor-joining method.

We focused on the IRE1α-Xbp1 pathway of UPR in this study and cloned the porcine *Xbp1 *gene (GenBank accession number FJ213449.1) as described in Materials and Methods. Porcine *Xbp1 *mRNA has two open reading frames (ORF) composed of 792 bp and 1137 bp which are alternatively produced by the IRE1α-mediated unconventional splicing (Figure 2A). Notably, 26 nucleotide sequences, splicing sites by IRE1α, within Xbp1 ORF and its flanking sequence are identical with those of the human *Xbp1 *sequences. When the secondary structures of pXbp1 sequence around the IRE1α cleavage sites were predicted using CentroidFOLD [18], the consensus cleavage sites were located in the loop-portion (Figure 2B and 2C). The amino acid sequences of the pXBP1 protein show significant homology with hXBP1 including the conserved basic leucine zipper (bZIP) region. The amino acid similarity was 89% between pXBP1 and hXBP1 (Figure 2D). Phylogenetic tree of XBP1 sequences from various organisms was generated using the neighbor-joining method. The phylogenetic tree showed that the pXBP1 is a closer match to the human XBP1 (Accession No. NM_005080, 85% amino acid identity) rather than that of a rat (Accession No. JC4857, 83% amino acid identity) or a mouse (Accession No. NP_038870, 80% amino acid identity) (Figure 2E).

### Unconventional splicing of the porcine Xbp1

**Figure 3:**
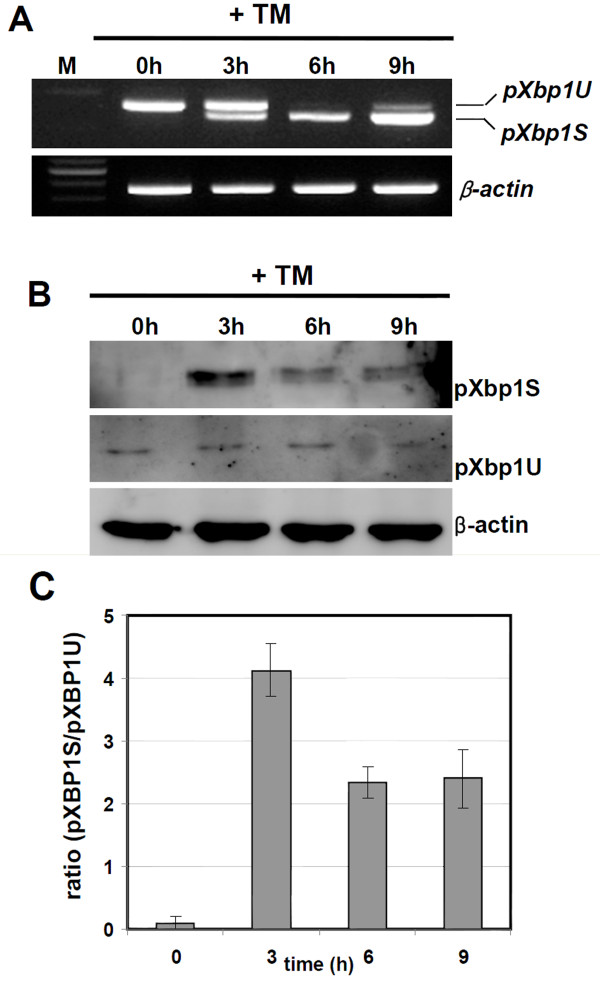
**ER stress induced the spliced *pXbp1 *mRNA and active pXbp1 protein**. Tunicamycin treatment induced spliced *pXbp1 *mRNA (A) and active pXbp1 protein (B) from 3 h. β-actin was used as the loading control. (C) Quantification of the Western blot analysis in (B). Data were presented as means ± SD from three independent experiments.

To elucidate whether IRE1α-mediated splicing of *pXbp1 *mRNA was triggered by ER stress like a human *Xbp1 *mRNA, PEF cells were treated with TM and performed the RT-PCR analysis. The spliced form of *pXbp1 *product (*pXbp1S*) was detected in the TM treated cells from 3 h. The spliced *pXbp1 *mRNA level was significantly increased during ER stress (Figure 3A). In the Western blot analysis, the expression level of the activated pXbp1 protein (Xbp1S) significantly up-regulated by the TM treatment from 3 h which is consistent with the RT-PCR analysis (Figure 3B). These results indicate that *pXbp1 *mRNA is processed upon ER stress and can be used as a major marker for UPR response in the porcine system.

### Nuclear localization of pXbp1 and ERAI vector system in PEF cells

**Figure 4:**
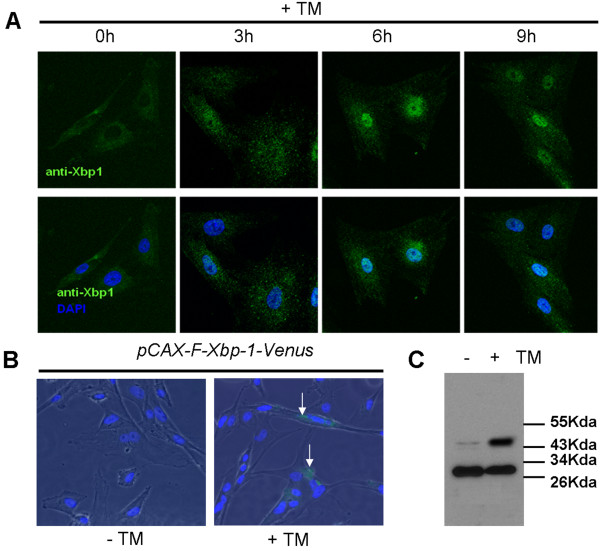
**Nuclear localization of pXbp1 and the ERAI vector system**. (A) pXbp1 was immunostained with the Xbp1 antibody in the TM treated PEF cells. pXbp1 protein was localized in cytoplasm under the normal condition but was predominantly translocated into the nuclei by the ER stress. (B) Detection of the ER stress-activated indicator (ERAI) by the green fluorescence (green) in the TM treated PEF cells (arrows). DAPI is used for marking the nuclei. (C) In the Western blot analysis with the ant-FLAG antibody, FLAG-tagged XBP1-venus fusion protein was found in PEF cells transfected with ERAI plasmids and treated with TM. The upper bands indicate the XBP1-venus fusion protein (45 kDa). The lower bands indicate the truncated XBP1 (30 kDa).

XBP1 belongs to a bZIP transcription factor. When cells are challenged with ER stress, activated XBP1 enters into the nucleus for activating its target genes [19]. When we tested the localization of XBP1 in the TM treated PEF cells by the immunostaining, pXBP1 was predominantly localized in the nucleus after 6 h (Figure 4A).

Recently Iwawaki et al [20] reported the ER stress-activated indicator (ERAI) vector system for monitoring ER stress with the green fluorescence protein (GFP). In the ERAI plasmid, human Xbp-1-Venus fusion construct contains the partial sequence of the human XBP1 and a GFP variant (Venus) sequence. Upon ER stress, 26 nucleotides of human *Xbp1 *are spliced out and the XBP1-Venus fusion protein is produced. To test whether ERAI works as an indicator for ER stress in the porcine system, we checked the GFP signal in the ERAI transfected PEF cells after TM treatment. We found that 20.5% of total cells showed GFP signal in the TM treated ERAI-PEF cells, not in TM non-treated ERAI-PEF cells (Figure 4B, arrows). This observation was consistent with the results of the Western blot analysis. The band of the XBP-1-venus fusion protein (45 kDa) was very strong under ER stress (Figure 4C). These results suggest that the ERAI system can be used for monitoring the activation of UPR in the porcine system.

### Porcine Xbp1 plays a role in ER stress-mediated cell death

**Figure 5:**
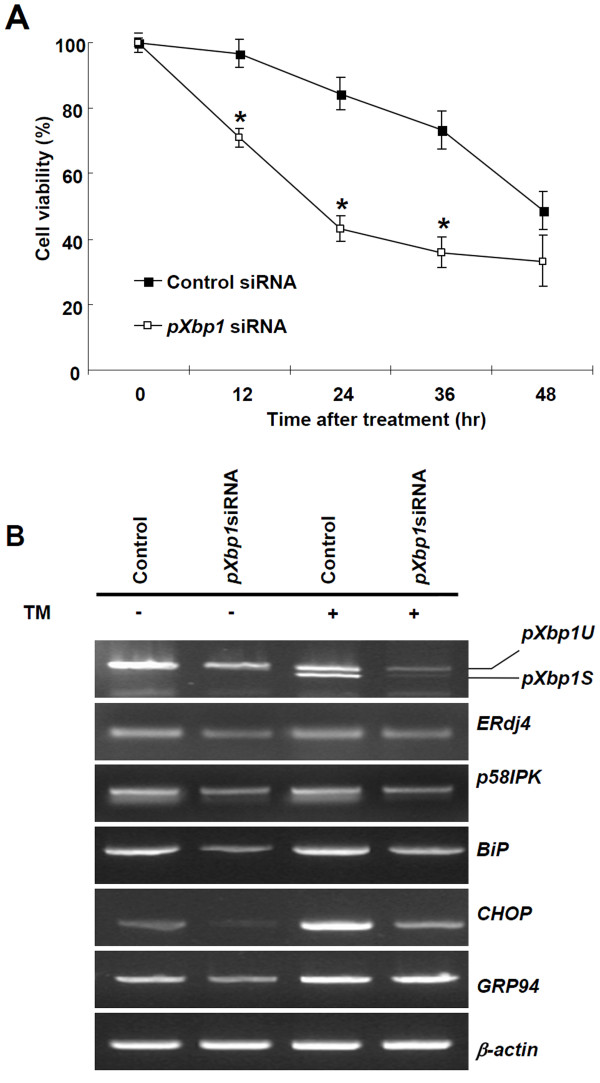
**Knock-down of *pXbp1 *increased ER stress-induced cell death and down-regulated expression of target genes**. (A) Cell viability of TM treated PEF cells was decreased by the knock-down of *pXbp1 *(open square) compared with the control siRNA (square). Data are presented as means ± SEM. *P < 0.05. (B) Knock-down of *pXbp1 *decreased expression of *ERdj4, p58IPK, BiP, CHOP*, and *GRP94 *target genes in the TM treated and non- treated PEF cells. β-actin was used as the loading control.

To investigate whether *pXbp1 *is involved in ER stress induced cell death, knock-down experiments were performed with *pXbp1 *siRNA. *pXbp1 *knock-down cells expressed only 20% of *pXbp1 *mRNA compared with the control siRNA transfected cells (Additional file 2, Fig. S2). In addition, we performed the Western blot analysis using the Xbp-1 antibody in *pXbp-1 *knock-down PEF cells. Active and inactive Xbp-1 proteins were significantly decreased in *pXbp1 *knock-down cells compared with the control siRNA transfected cells (Additional file 3, Fig. S3). *pXbp1 *knock-down cells displayed decreased cell viability compared with the control siRNA transfected cells (Figure 5A). To further support the role of *pXbp1 *in survival during ER stress, we compared the number of apoptotic cells in the control siRNA and *pXbp1 *siRNA transfected PEF cells by the TUNEL staining. In *pXbp1 *knock-down cells, TUNEL positive cells increased about 10-fold compared with the control (Additional file 4, Fig. S4). These results indicate that the knock-down of *pXbp1 *is more sensitive to ER stress-induced apoptosis, which is consistent with the published data in human myeloma cells [21].

Xbp1 activates its UPR target genes for maintaining the ER function and suppressing ER stress-induced cell death. The inhibition of *Xbp1 *activity by the dominant negative *Xbp1 *or knock-down *Xbp1 *markedly reduces the expression of Xbp1 dependent UPR target genes [6]. Thus, we investigated the expression of pXbp1 target genes during ER stress response. The knock-down *pXbp1 *significantly reduced the expression of *ERdj4 *and *p58IPK *both in the normal condition and TM treated PEF cells (Figure 5B). In addition, well-known UPR target genes, including *BiP, CHOP*, and *GRP94*, were also significantly induced by TM treated PEF cells, but reduced in the *pXbp1 *knock-down PEF cells (Figure 5B). These results suggest that pXbp1 dependent target gene expression might be required for maintaining cell viability in PEF cells under the ER stress condition.

### Porcine XBP1 as a UPR marker in the porcine system

**Figure 6:**
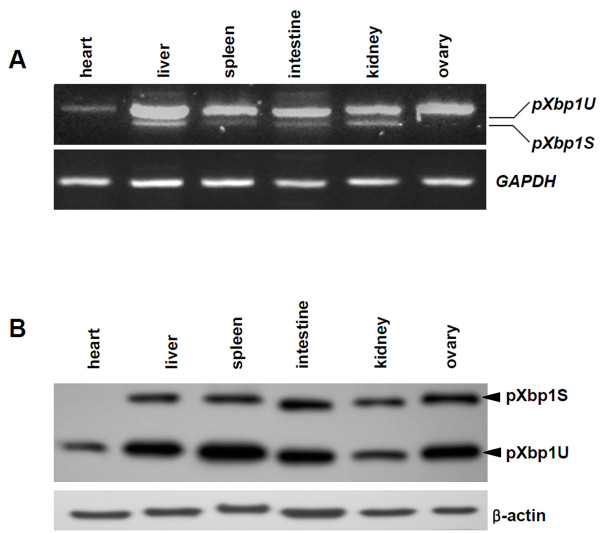
**Expression of *pXbp1 *mRNA and protein in adult organs**. Unspliced and spliced *pXbp1 *mRNA (A) and protein (B) was expressed in liver, spleen, intestine, kidney, and ovary.

Finally, we assessed the unconventional splicing pattern of *pXbp1 *as a UPR marker in the porcine system. Xbp1 is essential for development of liver and exocrine organs like the pancreas or salivary gland and holds a crucial role in immune systems and metabolic regulations [22-24]. To investigate expression of *pXbp1 *in adult porcine organs, we performed the RT-PCR and Western blot analysis with adult organs. Unspliced and spliced *pXBP1 *mRNA and protein was expressed in liver, spleen, intestine, kidney, and ovary (Figure 6A, B). In the absence of exogenously induced ER stress, the unconventional splicing of pXbp1 takes place in adult tissues. These results support that *pXbp1 *can be used for monitoring ER stress response and as a specific UPR marker in the porcine system.

## Discussion

The porcine model system is important to study human diseases because it shares similarities with humans in similar organ sizes, anatomy, and physiology [10]. Comparative genomic analysis showed that porcine genomic sequences are more similar to those of the human than to the genomic sequences of the mouse [25]. The UPR pathway is valuable for cellular homeostasis and animal development. Therefore, investigating the UPR pathway and monitoring ER stress in the model system is important to the study of pathogenic mechanisms that cause human diseases [1,3,26].

The results of this study show the UPR pathway in porcine system. As well as IRE1α-Xbp1 pathway, two ER transmembrane proteins, PERK and ATF6α, play important roles in the mammalian UPR. Upon UPR induction, ATF6α is released by BiP and exported to the Golgi, where it is cleaved by S1P proteases [5,27]. Cleaved ATF6α acts as a transcriptional activator that can increase the expression of ER chaperones and *Xbp1 *[5]. In this study, we found that three UPR components (IRE1α, PERK, and ATF6α) are activated by ER stress in porcine cells (Figure 1D and 1E). Among these three UPR sensors, IRE1α-Xbp1 pathway plays a central role in ER stress response by the transcriptional regulation of genes involved in ER function. The amino acid sequence comparison and the phylogenetic analysis showed that pXbp1 has a high similarity to human Xbp1. In addition, the consensus sequence (CAGCAC/G) in *Xbp1 *mRNA of IRE1α target site is conserved between pigs and human (Figure 2). These results suggest that the IRE1α-Xbp1 pathway is evolutionary conserved in mammals.

Conventional splicing is catalyzed by the spliceosome for removing an intron within a primary transcript [28]. In contrast, *Xbp1 *pre-mRNA is cleaved by IRE1α endonuclease-mediated unconventional splicing and this process is independent of the spliceosome [5,29]. Unconventional splicing of *Xbp1 *mRNA leads to the production of an active transcription factor and induction of adaptive genes in the mammalian UPR. We showed that pXbp1 was also cleaved by unconventional splicing during ER stress response (Figure 3). Spliced *pXbp1 *mRNA was detected in the TM treated cells from 3 h and gradually increased until 9 h. In contrast, the protein level of active pXbp1s peaked within 3 h and slightly decreased from 6 h. During ER stress response, activated PERK pathway attenuates global protein translation by phosphorylating the eIF2α [30]. We also detected that active PERK increased from 6 h in the TM treated cells (Figure 1D, E). Therefore, the translation of spliced and active *Xbp1 *mRNA may be attenuated by the PERK-eIF2α pathway.

In this study, we revealed that pXbp1 suppression is involved ER-mediated cell death through the regulation of target gene expression, such as ERdj4, p58IPK, and BiP. In addition, the ER stress-induced expression of CHOP, a pro-apoptotic transcription factor, was also reduced in the pXbp1 knockdown cell (Figure 5). The CHOP promoter contains ER stress response element (ERSE) and amino-acid-regulatory element (AARE). All three UPR transcription factors, ATF4, ATF6 and XBP1, can bind these promoter regions and regulate the transcription of CHOP [31]. The expression of CHOP is reduced in Xbp1 knockout MEF cell line [6] and in *pXbp1 *knock-down porcine cells (Figure 5). These results indicate that pXbp-1 may be involved in the regulation of CHOP expression. However, PERK-ATF4 and ATF6α are known as dominant regulators for the CHOP expression rather than XBP1 [32,33] and all three UPR pathways are required for the maximal induction of CHOP [34].

Studies in the mouse model show that the IRE1α-XBP1 pathway is essential for the liver and heart development and the function of secretion in pancreatic exocrine and plasma cells [22-24,35]. The embryonic fibroblasts from XBP1-deficient mouse show defects in adipogenesis [36]. We have investigated expression of the inactive and active isoforms of pXBP1 in various porcine tissues. While unspliced *pXbp1 *was expressed in all examined tissues, spliced *pXbp1 *was strongly expressed in liver and kidney (Figure 6A). Furthermore, active pXbp1s protein was detected in all examined tissues except heart (Figure 6B). This result indicates that pXbp1 plays a role in porcine tissues like other animals.

## Conclusion

In this study, we show that unconventional splicing of *pXbp1 *mRNA is evolutionary conserved and pXbp1 is involved in ER-stress mediated apoptosis. In the adult porcine organs, spliced *pXbp1 *was expressed. These results suggest that pXbp1 can be used to monitor physiological and pathological ER stress in the porcine model system.

## Methods

### Cell culture and ER stress condition

Porcine embryo fibroblast (PEF) cells derived from 25-26 days of fetal pigs were cultured in Dulbecco's modified Eagle medium supplemented with 10% heat inactivated fetal bovine serum (FBS), 1% NEAA, 0.1% Gentamicine. PEF cells were maintained at 37°C in a humidified atmosphere of 5% CO_2_. In order to induce ER stress, PEF cells seeded in a 6-well dish were treated with Tunicamycin (TM, 2 μg/ml in DMSO) and harvested at 3 h, 6 h, and 9 h. Animal care and all experiments were conducted in accordance with KRIBB Guidelines for the Care and Use of Laboratory Animals and all experiments were approved by the institutional review board (KRIBB Institutional animal care and use committee/KRIBB-IACUC).

### Cell viability assay

Cell viability was determined by 3-(4,5-dimethylthiazol-2-yl)-2,5-diphenyl tetrazolium bromide (MTT) assay using the CellTiter 96 Aqueous One kit (Sigma, St. Louis, MO, USA) according to the manufacturer's instructions.

### Cloning of the porcine Xbp1 cDNA and phylogenetic analysis

To isolate porcine *Xbp1*, we searched the GenBank EST database with the BLAST (tblastn) program using the human *XBP1 *(GenBank accession number NM_005080) as the query sequence. LVRM100070G04, a *Sus scrofa *mRNA clone expressed in liver (GenBank accession number AK233029.1), showed high homology with the human *XBP1 *mRNA sequence. Oligonucleotide primers (pXBP1-F1, pXBP1-R1) were designed to clone the complete sequence of porcine *Xbp1 *mRNA (Figure 2A; Additional file 5, Table S1). PCR reaction was prepared with porcine cDNA generated from PEF cells, 1 pM of each primer, and 1X *AccuPower*^® ^PCR premix (Bioneer, Daejeon, Korea). PCR Amplification was performed with 30 cycles of 94°C for 30 sec, 58°C for 30 sec, and 72°C for 2 min using GeneAmp PCR system 9700 (Applied Biosystems, Carlsbad, CA, USA). The cloned 1.7 kb PCR product was subcloned into the pGEM-T easy vector (Promega, Madison, WI) and was sequenced using an ABI automated sequencer (Applied Biosystem, Carlsbad, CA, USA). Homology searches for the cloned cDNA sequence and deduced amino acid sequences were performed by BLAST programs. Analysis of the primary structure of predicted proteins were performed by the SMART (Simple Modular Architecture Research Tool) at ExPASy Proteomics Server. Phylogenetic analysis was performed using ClustalW (http://www.ch.embnet.org). GenBank accession numbers for human XBP1: NM_002256, mouse Xpb-1: NM_178260, rat Xpb-1: NM_181692; bovine Xpb-1: XM_867473, Xenopus BK006297; zebrafish, EF690279; *Drosophila*, EF690279; *C. elegans *EF690279.

### RNA preparation and reverse transcription-PCR analysis

The total RNA of PEF cells were extracted with Trizol reagent (Invitrogen, Carlsbad, CA, USA) and were further purified by On-Column RNase-free DNase digestion (Qiagen, Hilden, Germany) to remove possible genomic DNA contamination. cDNA was synthesized by using Superscript II^® ^Reverse Transcription system (Invitrogen, Carlsbad, CA, USA). For RT-PCR, *AccuPower*^® ^PCR premix (Bioneer, Daejeon Korea) was used with the set of primers. Primer sequences are in Additional file 5 (Table S1). Amplification conditions were as follows: a single cycle of 94°C for 5 min followed by 30 cycles of 94°C for 30s, 58°C for 30s, and 72°C for 30s, and the final single cycle of 72°C extension for 7 min. PCR products were separated by electrophoresis in the 2% agarose gel.

### Immunostaining

PEF cells were cultured on a gelatin-coated coverslip of up to 40% confluence. Cells were fixed in PBS contained 4% (w/v) formaldehyde for 30 min at room temperature (RT) and washed in PBS with 0.05% Tween 20 (PBST). After the incubation for 30 min in PBS containing 1% Triton X-100, the samples were blocked in PBST with 2% BSA for 1 h at RT, and incubated for 1.5 h at 37°C with a rabbit anti-Xbp1 antibody (1:200, Santa Cruz Biotechnology, Santa Cruz, CA, USA). Then, the cells were washed in PBST, incubated with a secondary antibody (1:200) for 1 h at 37°C, washed in PBST for 15 min four times, and mounted on Poly-prepslide glass (Sigma Aldrish, MO, USA) with a mounting media containing DAPI (Vectashield; Vector Laboratories, Burlingame, CA, USA). Samples were observed with a Karl Zeiss Axiovert 200 M fluorescence microscope equipped with an Apotome apparatus. Images were captured digitally using different filter sets and merged using AxioVision (v4.5) and Adobe Photoshop software (v7.0).

### Western blot analysis

Protein preparation, SDS-PAGE, and immunoblotting were performed as previously described [37]. KDEL (1:1000, Assay designs, Ann Arbor, MI, USA), phospho-PERK, PERK, IRE1α, ATF6α from (1:800, Santa Cruz Biotechnology, Santa Cruz, CA, USA), phospho-IIRE1α from (1:800, Abcam, Cambridge, MA, USA), ATF6α (1:1000, Santa Cruz Biotechnology, Santa Cruz, CA, USA), XBP1 (1:1000, New England Biolabs, Ipswich, MA, USA) or β-actin (1:2000, Abcam, Cambridge, MA, USA) primary antibodies and horseradish peroxidase (HRP)-conjugated secondary antibodies (Santa Cruz Biotechology, Santa Cruz, CA, USA) were used.

### RNA interference

Customized Stealth siRNA duplex oligonucleotides against *pXbp1 *(Genbank accession no. FJ213449.1) were synthesized by Invitrogen (Carlsbad, CA, USA). The sequences were as follows: sense 5'-UGAAGAGUCAACACCGUCAGA AUCC-3', antisense 5'-GGAUUCUGACGGU-GUUGACUCUUCA-3'. BLOCK-It Fluorescent Oligo (Invitrogen, Carlsbad, CA, USA) was used as the control. Before transfection, cells were plated at 50% confluency in Dulbecco's modified Eagle medium supplemented with 5% fetal bovine serum without antibiotics and incubated overnight. Transfections were performed with Lipofectamine™ RNAiMAX (Invitrogen, Carlsbad, CA, USA) as directed by the manufacturer. Transfection efficiencies were checked by the fluorescent-labeled control siRNA. The final concentration of the siRNA was 100nM. After 24 h incubation with siRNA, RT-PCR and cell viability assay were performed.

### Statistical analysis

All experiments were repeated at least three times and the data was presented as the mean and error bar (± S.E.M.). The statistical significance was tested by Microsoft Excel-based application for the student t-test statistical analysis. A *p*-value of < 0.05 was considered to be significant.

## Authors' contributions

KSL, DBK and KY designed the experiments. JYZ, KSL, JSK, and BSS performed the experiments. DIJ supplied materials. KSL and KY analyzed and interpreted data. JYZ, KSL and KY wrote the manuscript. All authors read and approved the final manuscript.

## Supplementary Material

Additional file 1 **Figure S1 Tunicamycine triggers ER stress-mediated cell death in PEF cells**. Cell morphology and caspase-3 activity Click here for file

Additional file 2 **Figure S2 Knock-down of pXbp1 by the pXbp1 siRNA in PEF cells**. Expression levels of pXbp1 mRNA was measured by RT-PCR Click here for file

Additional file 3 **Figure S3 Knock-down of pXbp1 by the pXbp1 siRNA in PEF cells**. Expression levels of pXbp1 protein was measured by the Western blot analysis using the anti-XBP-1 antibody Click here for file

Additional file 4 **Figure S4 pXbp1 Knock-down enhances ER stress-mediated cell death**. Cell death was analyzed by TUNEL staining in PEF cells Click here for file

Additional file 5 **Table S1 PCR Primers used in this study**. A description of PCR primers used in this study Click here for file
