# Supplementary material for: Functional characterization of the ER stress induced X-box-binding protein-1 (Xbp-1) in the porcine system
Source: BMC Mol Biol. 2011 May 24;12:25. doi: 10.1186/1471-2199-12-25 (PMC3112107; doi:10.1186/1471-2199-12-25)
Supplement: Additional file 1 — Figure S1 Tunicamycine triggers ER stress-mediated cell death in PEF cells. Cell morphology and caspase-3 activity [file 1471-2199-12-25-S1.PDF]

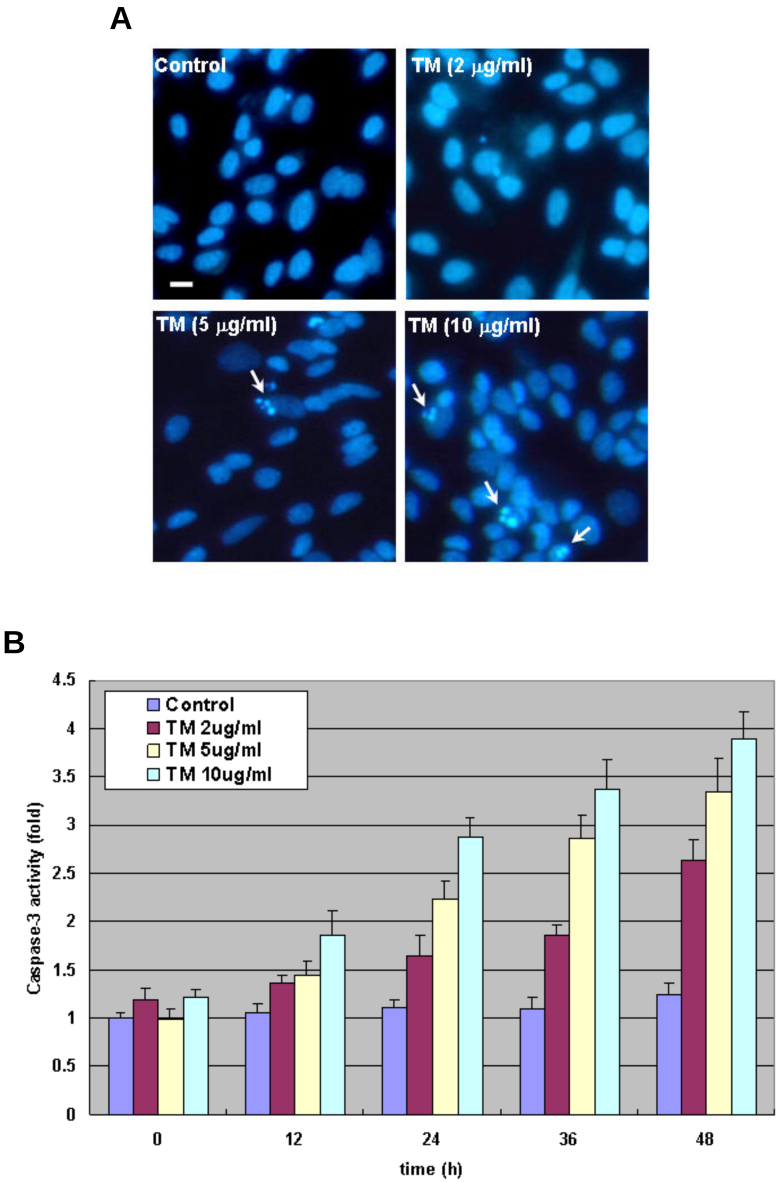

Figure S1. Tunicamycine triggers ER stress-mediated cell death in PEF cells (A) Cells were stained with Hoechst 33258 staining. Dead cells were identified by morphological changes such as nuclei fragmentation (arrows). Scale bar: 10 μm. (B) Caspase-3 activities increased in the TM treatment by time and dose-dependently. Data were presented as means ± SD from three independent experiments.
