# Supplementary material for: Functional characterization of the ER stress induced X-box-binding protein-1 (Xbp-1) in the porcine system
Source: BMC Mol Biol. 2011 May 24;12:25. doi: 10.1186/1471-2199-12-25 (PMC3112107; doi:10.1186/1471-2199-12-25)
Supplement: Additional file 2 — Figure S2 Knock-down of pXbp1 by the pXbp1 siRNA in PEF cells. Expression levels of pXbp1 mRNA was measured by RT-PCR [file 1471-2199-12-25-S2.PDF]

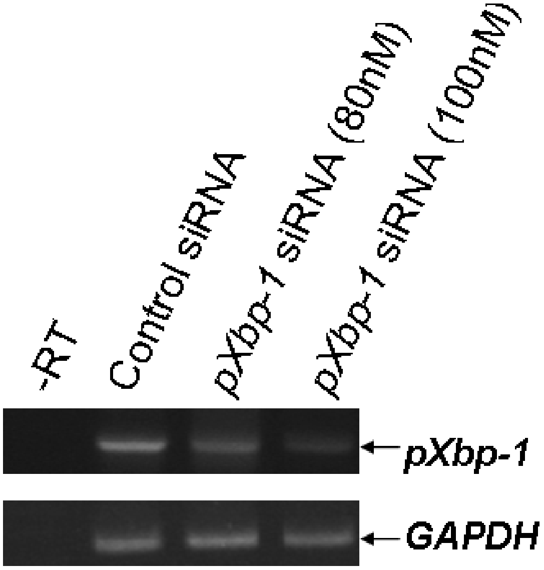


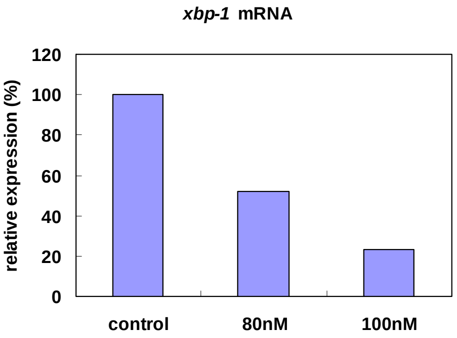


**Figure S2. Knock-down of *pXbp1* by the *pXbp1* siRNA in PEF cells.** PEF cells were transfected with the control siRNA or *pXbp1* siRNA and expression levels of *pXbp1* mRNA was measured by RT-PCR. GAPDH was the loading control.
