# Supplementary material for: Functional characterization of the ER stress induced X-box-binding protein-1 (Xbp-1) in the porcine system
Source: BMC Mol Biol. 2011 May 24;12:25. doi: 10.1186/1471-2199-12-25 (PMC3112107; doi:10.1186/1471-2199-12-25)
Supplement: Additional file 4 — Figure S4 pXbp1 Knock-down enhances ER stress-mediated cell death. Cell death was analyzed by TUNEL staining in PEF cells [file 1471-2199-12-25-S4.PDF]

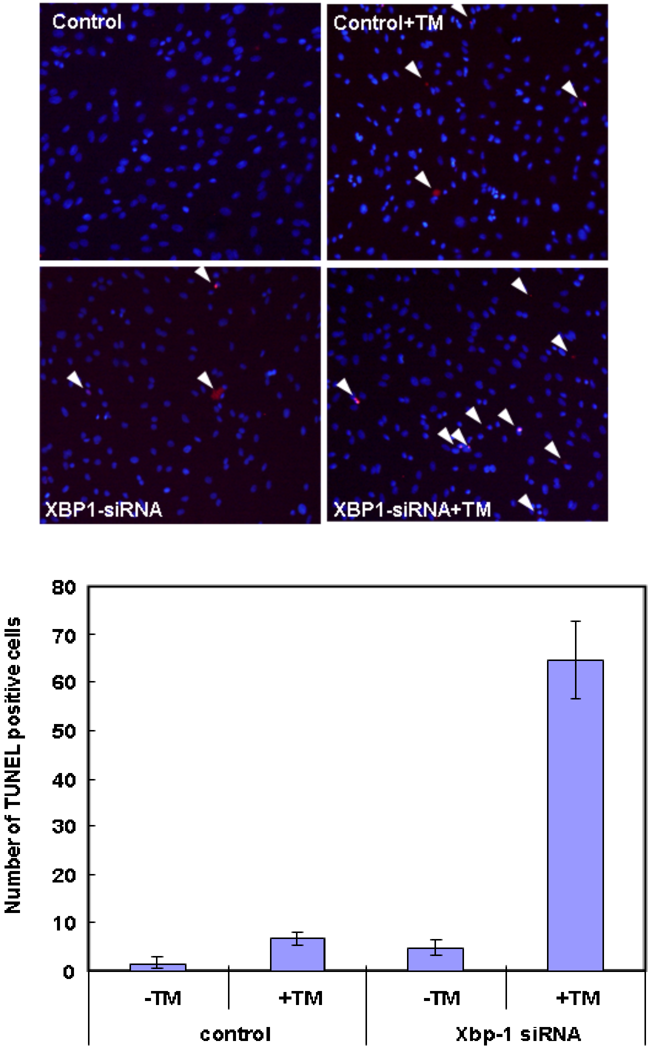

Figure S4. pXbp1 Knock-down enhances ER stress-mediated cell death. Cell death was analyzed by TUNEL staining with DAPI in PEF cells. Tunicamycin (TM) was treated for 12h.
