# Supplementary material for: Functional characterization of the ER stress induced X-box-binding protein-1 (Xbp-1) in the porcine system
Source: BMC Mol Biol. 2011 May 24;12:25. doi: 10.1186/1471-2199-12-25 (PMC3112107; doi:10.1186/1471-2199-12-25)
Supplement: Additional file 5 — Table S1 PCR Primers used in this study. A description of PCR primers used in this study [file 1471-2199-12-25-S5.PDF]

Table S1. PCR Primers used in this study

GenBank Accession No.	Gene name	Primer sequence	Product size (bp)	Tm (℃)
FJ213449.1	pXbp-1	**Forward:** GGCCCCCAAAGTACTGCTTC<br>**Reverse:** TGGAGAAAGCACCTTCCAAAA	1750	60
XM_003134777.1	ERdj4	**Forward:** TCCAGACACGCCAGGATGGT<br>**Reverse:** GCCCAAGGGCAGGTAGAGAAA	522	60
NM_001190184.1	p58IPK	**Forward:** TGGAGCCTGACAATGTGAATGC<br>**Reverse:** TCGCCGTCGTCGAACTTCTT	382	59
FJ213449.1	pXbp-1s	**Forward:** GGCAGAGACCAAGGGGAATG<br>**Reverse:** GGGTCGACTTCTGGGAGCTG	263	58
X92446.1	GRP78/BiP	**Forward:** CCAACTGTTACCATCAAGGTCTAT<br>**Reverse:** CTCATCTTTGTCTGCTGATTCCTC	586	60
NM_001144845.1	GRP94	**Forward:** AAGGAGAATCGTGAAGCAGTTGAG<br>**Reverse:** TTGCTCTGTGTCTTCTGTGGTGTC	517	61
Y09136.1	CHOP	**Forward:** AAAGCAGAGCCTAATCCAGCC<br>**Reverse:** CTGCTTGAGCCGTTCGTTCT	450	60
AF017079	GAPDH	**Forward:** GGGCATGAACCATGAGAAGT<br>**Reverse:** AAGCAGGGATGATGTTCTGG	230	55
